# Development of microfluidic ELISA for measuring humoral responses to clostridial antigens in vaccinated cattle

**DOI:** 10.1016/j.jim.2025.113900

**Published:** 2025-06-23

**Authors:** Joo Youn Park, Amelia Woolums, Robert Wills, Rhonda Vann, Keun Seok Seo

**Affiliations:** a Department of Comparative Biomedical Sciences, College of Veterinary Medicine, Mississippi State University, Mississippi State, MS 39762, USA; b Department of Pathobiology and Population Medicine, College of Veterinary Medicine, Mississippi State University, Mississippi State, MS 39762, USA; c Department of Animal and Dairy Sciences, College of Agriculture and Life Sciences, Mississippi State University, Mississippi State, MS 39762, USA

**Keywords:** Microfluidic ELISA, Clostridial toxins, Cattle vaccination, Antibody titers

## Abstract

Clostridial diseases significantly threaten livestock health, particularly in cattle, underscoring the need for effective vaccination strategies. This study develops and optimizes a microfluidic-based enzyme-linked immunosorbent assay (ELISA) for the rapid (< 1 h) and cost-effective measurement of IgG antibody levels against various clostridial toxins in cattle vaccinated with a multivalent clostridial vaccine. This assay requires only 5 μL of sample and reagent volume, demonstrating high repeatability and reproducibility with coefficient of variation (CV) values ranging from 0.1 % to 8.5 % across all tested clostridial antigens. The Limit of Detection (LOD) of the assay ranged from 1:150 to 1:800, allowing for sensitive detection of antibody levels. For *Clostridium perfringens* ε toxin, antibody titers were measured using a commercial kit, while microfluidic ELISA was applied to assess titers against tetanus toxoid, *Clostridium septicum* α toxin, *Clostridium novyi* type B toxins, and *Clostridium sordellii* toxins. Significant increases in IgG antibody levels were observed for *C. perfringens* ε toxin and tetanus toxoid following both primary and booster vaccine doses, peaking by day 42. Antibody titers against *C. septicum* α toxins and *C. novyi* type B toxins increased after the primary dose, peaking at day 42, while no booster effect was seen for *C. sordellii*. These findings highlight the utility of microfluidic ELISAs as a practical and efficient tool for assessing humoral immune responses to clostridial toxins in vaccinated cattle, with potential application for the herd level disease surveillance and vaccine efficacy assessment.

## 1. Introduction

Clostridia are Gram-positive, anaerobic, spore-forming bacteria often found in the soil and manure or in contaminated feedstuffs (Cruz-Morales et al., 2019). Their spore-forming nature allow them to persist in various environments for extended periods (Shen et al., 2019), and they are often present as commensals in the guts of healthy cattle (Lopetuso et al., 2013). Although the prevalence and outbreak rate of clostridial diseases in cattle are relatively low compared to other pathogens, they still can cause sudden death and other fatal outcomes, posing significant risks. Serious clostridial infections in cattle are caused by toxins produced by *Clostridium perfringens*, *Clostridium septicum*, *Clostridium novyi*, *Clostridium sordellii*, and other pathogens in this genus (Gohari and Prescott, 2022). Since treating clostridial disease in cattle is often ineffective once clinical signs appear, clostridial vaccines are the most effective control measure to prevent clostridial outbreaks and reduce economic losses of these diseases in cattle (Uzal, 2012; Abdolmohammadi Khiav and Zahmatkesh, 2021).

The measurement of antigen-specific immunoglobulin G (IgG) concentration is a clinically important indicator for humoral responses to vaccination, immunogenicity and vaccine potency. However, commercially available assays for this purpose in cattle remain limited, primarily targeting *C. perfringens* toxins. Typically, standard method for evaluating the potency of clostridial vaccine, such as *in vitro* cytotoxicity assays using cell lines, or *in vitro* and *in vivo* toxin neutralization tests, are time consuming, costly, and impractical for herd-level population studies (Crichton et al., 1986; Aldape et al., 2006; Woolums et al., 2011; Moreira et al., 2016; Silva et al., 2018; Augusto de Oliveira et al., 2019). Therefore, there is a need for a more efficient and specific method to accurately measure clostridial antigen-specific immune responses in cattle, enabling better evaluation of vaccine responses at both the individual and herd levels.

In recent years, microfluidic technology has been continuously and extensively developed for application in various immunoassay platforms (Vashist et al., 2015; Berlanda et al., 2021; Wu et al., 2022; Bhuiyan and Shim, 2024). Among these, microfluidic microplate-based immunoassays, including enzyme-linked immunosorbent assays (ELISA), have gained attention as next generation immunoassay platforms. This approach adapts the traditional 96-well plate format into a microfluidic channel configuration, enhancing assay performance (Kai et al., 2012; Jung et al., 2015; Uddin et al., 2021). Several studies have shown that these microfluidic microplates are highly sensitive and rapid, using 20 times less volume compared to conventional ELISA (Kai et al., 2012; Uddin et al., 2021). While conventional ELISAs have been widely and extensively used in both research and clinical applications, they have some drawbacks such as long assay time (4–24 h), labor-intensive procedures, and large sample/reagent volumes (~100 μL), making them less feasible for large-scale immunological studies in cattle.

To address these limitations of standard *in vitro* and *in vivo* tests, as well as conventional ELISA, we developed and optimized an ELISA to measure IgG titers against various clostridial toxin antigens using specialized microplates, specifically, microfluidics-based microplates. We demonstrate here that this assay reduces sample and reagent volume needed, and assay time, with high reproducibility of results. Using this optimized microfluidic ELISA, we subsequently assessed serum antibody titers in cattle after the first and booster vaccinations with a multivalent clostridial vaccine.

## 2. Materials and methods

### 2.1. Ethics approval

All animal experiments in this study were approved and conducted under the supervision by the Mississippi State University Institutional Animal Care and Use Committee (IACUC protocol #20–124).

### 2.2. Control serum for ELISA optimization

Two mature mixed-breed beef steers, previously vaccinated over several years with multivalent clostridial vaccines, received a booster dose (2 mL) of the commercially available multivalent vaccine, BOVILIS^®^ CAVALRY^®^ 9 (Merck & Co. Rahway, NJ, USA), a bacterin-toxoid marketed to aid in the prevention of disease caused by *C. perfringens* types C and D, *C. tetani, C. septicum, C. novyi* type B, *C. sordellii*, *C. chauvoei*, and *C. haemolyticum*, according to the manufacturer’s label. Sera collected on day 0 (before booster), as well as days 14 and 28 after booster, were used as positive controls for ELISA optimization. Additionally, sera collected from six 4-month-old beef calves that had not been vaccinated against clostridial agents were used as negative controls for ELISA optimization.

### 2.3. Clostridial vaccination and blood sampling of cattle

A total of 12 mixed-breed beef steers and heifers (6 steers and 6 heifers), none of which had been previously vaccinated against clostridial agents, were included in the study. These calves were born between February 8 and March 6, 2021, with an average age (range) of 112 days (95–121 days) on the day of the first vaccination. The animals were vaccinated subcutaneously in the cervical region with 2 mL of BOVILIS^®^ CAVALRY^®^ 9 on day 0, followed by a booster administered in the opposite cervical regions on day 21. Blood samples was collected on day 0 (before vaccination), 14, 21 (before booster), 42, and 56. Serum samples were then isolated from these blood samples and stored at −20^o^ C until further use.

### 2.4. Microfluidic multiwell microplates

**Fig. 1.**
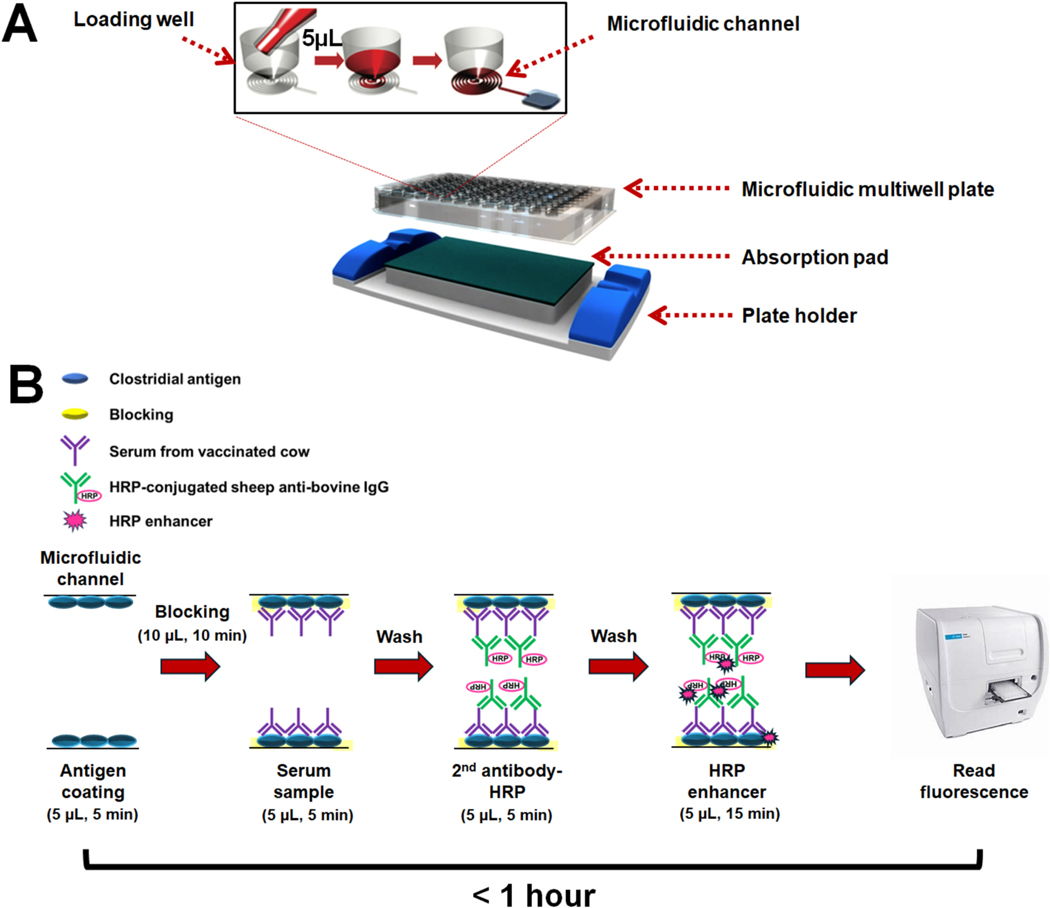
(A) Schematic illustration of microfluidic multiwell microplate. (B) Overview of the steps and process timeline for the microfluidic ELISA assay.

The microfluidic microplate used in the development of the ELISAs described here (Optimiser^™^, MiCo BioMed Co., Blue Ash, Ohio, USA) features a spiral microfluidic channel at the base of each well, serving as a specialized reaction chamber (Fig. 1A). This plate design improves assay efficiency by decreasing the required volume of samples and reagents. Assays using these plates can often be completed more rapidly that ELISAs using traditional multiwell plates, and sensitivity is often improved (Kai et al., 2012). Reagents are loaded into each well, where they are drawn into the microchannel through capillary action. Any excess reagent is then drawn into an absorbent pad positioned beneath each well. All reactions occur within the microchannel itself. The spiral microfluidic channel design reduces diffusion distances and increases the surface-to-volume ratio compared to traditional microplates. This configuration appears to facilitate rapid assay kinetics and decreases required sample and reagent volume, which can make the assay less time-consuming and less expensive to run. A schematic overview of microfluidic ELISA procedure is summarized in Fig. 1B.

### 2.5. Measurement of serum antibodies to C. perfringens ε toxin

To confirm the suitability of control serum samples for optimizing the microfluidic ELISA for other 4 clostridial antigens including tetanus toxoid, *C. septicum α* toxins, *C. novyi* type B toxins, and *C. sordellii* toxins, IgG antibody titers against *C. perfringens* ε toxin in both positive and negative control sera were measured using a commercial ELISA kit (BioX K222, BioX Diagnostics, Rochefort, Belgium) according to the manufacturer’s instruction. Serum antibody titers against *C. perfringens* ε toxin was also measured in 12 vaccinated cattle using the same kit. The assay is a competitive ELISA, with results reported as percent inhibition of the competing standard. Higher percent inhibition values indicate high antibody titers. Each sample was tested in triplicate.

### 2.6. Optimization and development of ELISAs for various clostridial toxin antigens using microfluidic multiwell microplates

For the optimization and development of microfluidic ELISA, four different clostridial toxin antigens were used. Tetanus toxoid (*C. tetani,* 582,231) was obtained from Sigma-Aldrich, while culture filtrates containing *C. septicum* α toxin (CVB-DAT-5152), *C. novyi* type B toxins (CVB-DAT-0141), or *C. sordellii* toxin (CVB-DAT-5116) were sourced from Animal and Plant Health Inspection Services, United State Department of Agriculture (APHIS-USDA). The 96 well-microfluidic multiwell microplates were used.

During the optimization process, several factors were systematically adjusted to identify the optimal conditions for detecting IgG antibodies against each clostridial antigen, including the antigen dilution factor, serum dilution factor, and secondary antibody dilution factor. Positive and negative control serum samples, with their suitability confirmed using a commercial ELISA kit for *C. perfringens* ε toxin, were used to validate the assay.

To optimize antigen coating conditions, tetanus toxoid was tested at 5 ng, 25 ng, or 50 ng per well (Fig. S1A). For the other clostridial toxins, culture filtrates were used at dilution of 1:10, 1:20, and 1:50 (Fig. S2A-S4A). To determine the most effective concentration for serum use, positive and negative control serum samples were serially diluted 2-fold from 1:50 to 1: 400 and tested (Fig. S1B-S4B). Different concentrations of horseradish peroxidase (HRP) conjugated sheep anti-bovine IgG1 (Bethyl Laboratories, San Diego, California, USA), were also evaluated, with serial dilutions ranging from 1:10,000 to 1:160,000 for tetanus toxoid and from 1:5000 to 1:40,000 for the other toxins (Fig. S1C–4C). The optimal conditions were selected based on achieving the highest fluorescence intensity ratio between the positive and negative control serum samples.

The ELISA procedure began by coating the microfluidic multiwell microplates with 5 μL of tetanus toxoid (5 μg/mL) or the other clostridial toxins (1:20 dilution) for 5 min at room temperature (RT). Following the coating, the wells were washed with phosphate-buffered saline (PBS) and then blocked with 10 μL of an OptiBlock blocking solution (MiCo BioMed Co.) for 10 min at RT. After another wash with PBS, 5 μL of diluted serum (1:50 in PBS) was added to each well in triplicate, with positive and negative control sera included for each plate. The serum samples were incubated for 5 min at RT. After this incubation, the wells were washed with PBS, and 5 μL of HRP-conjugated secondary antibody was added to each well, with incubation for 5 min at RT. After the second wash with PBS, 5 μL of OptiGlow solution (MiCo BioMed Co.) was added to the wells and incubated for 15 min at RT. The fluorescence readings were taken at an excitation wavelength of 530 nm and emission at 590 nm using a Cytation 5 cell imaging multimode reader (Agilent, Santa Clara, CA, USA).

For the optimization process, results from the positive and negative control sera (*n* = 2 positive, *n* = 6 negative) were expressed as the mean ± standard deviation (SD) of the fluorescence intensity readings.

### 2.7. Assay performance evaluation: Reproducibility and limit of detection (LOD)

The repeatability and reproducibility of each microfluidic ELISA was evaluated using pooled positive and pooled negative control sera. Each serum was tested in triplicate with optimized conditions on the same day and over three different days. The intra-assay coefficient of variance (CV) was calculated to assess the repeatability within a single experiment, while the inter-assay CV was calculated to assess reproducibility across different experimental runs (*i.e.*, on different days). CV was determined as the standard deviation (SD) divided by the mean and expressed as a percentage.

The LOD for IgG antibody titers against each clostridial toxin antigen was determined under optimized conditions for each ELISA. The LOD cut-off was calculated based on the mean fluorescence intensity and SD of the pooled negative control sera, which were diluted 1:50. Specifically, the LOD was defined as the lowest antibody dilution at which the fluorescence intensity of the positive control serum exceeded the mean of the negative control by at least 3 × SD.

### 2.8. Measurement of serum antibodies to various clostridial toxin antigens

Serum antibody titers against various clostridial toxin antigens were measured in 12 vaccinated cattle at day 0 (before vaccination), 14, 21 (before booster), 42, and 56 after vaccination using an optimized microfluidic ELISA. Antibody levels for each sample were expressed as a percentage relative to the negative control serum, which was set at 100 %. Each sample was tested in triplicate.

### 2.9. Statistical analysis

The effect of sex and day of sampling on titers to *C. perfringens* ε toxin, tetanus toxoid, *C. novyi* type B toxin, *C. septicum* α toxin, and *C. sordellii* toxin was assessed using separate linear mixed models with the MIXED procedure in SAS for Windows v9.4 (SAS Institute, Inc., Cary, NC). Initial models included sex, day of sampling, and their interaction as fixed effects. Insignificant terms were sequentially removed starting with the sex-day of sampling interaction. As sex had no significant effect, it was also excluded from each of the final models. A repeated statement using a spatial power covariance structure was used to account for the repeated testing of cattle. In the case of a significant effect of day of sampling, pairwise comparisons were made between day 0 and each of the other days and between day 21 and the following testing days, day 42 and day 56. Visual assessment of residuals was used to ensure the models had met the assumptions of homoscedasticity and normality. An alpha level of 0.05 was used to denote statistical significance.

## 3. Results

### 3.1. Validation of positive and negative control sera

**Fig. 2.**
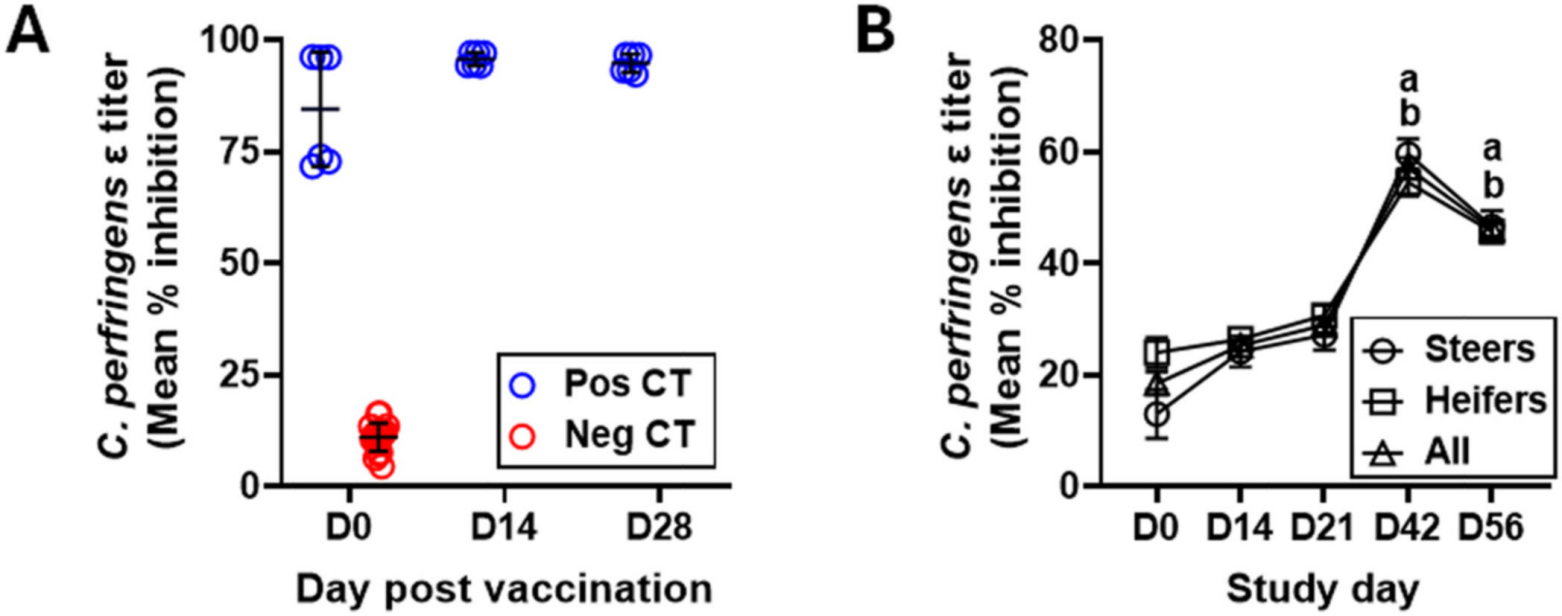
Measurement of IgG titers against *Clostridium perfringens* ε toxins using a commercial ELISA kit. (A) Titers of positive (*n* = 2) and negative (*n* = 6) control sera. (B) Serum from calves (steers, heifers, and all calves) vaccinated with a multivalent clostridial vaccine on day 0 and day 21. Titers increased after the initial dose on day 0 and again after the booster dose on day 21. The superscript ‘a’ indicates statistically significant differences (*p <* 0.05) on days 14, 21, 42, and 56, compared to day 0, while the superscript ‘b’ denotes significant differences on days 42 and 56, compared to day 21. Statistical significance was evaluated by separate linear mixed models with the mixed procedure.

To validate the suitability of the positive and negative control sera for optimizing the ELISAs, we measured antibody titers expressed as the percentage of inhibition using a commercial ELISA for *C. perfringens* ε toxin. In the positive control steers, which had been vaccinated multiple times over several years, serum antibody titers to *C. perfringens* ε toxin were significantly higher than those negative control serum (Fig. 2A). The average percent inhibition for each steer was as follows: Steer 1 — day 0: 96.2 %, day 14: 97.1 %, and day 28: 96.7 %; Steer 2 — day 0: 72.8 %, day 14: 94.4 %, and day 28: 92.3 %. In contrast, the serum antibody titers to *C. perfringens* ε toxin in the negative control sera from six calves that had not been vaccinated against clostridial agents were very low with an average percent inhibition of serum for each calf measured between 4.4 % and 16.3 % (Fig. 2A). Based on these results, serum samples from day 14 of both positive control steers and serum samples from six negative control calves were used for optimization of the microfluidic ELISA in this study.

### 3.2. Measurement of antibody titer to C. perfringens ε toxin using a commercial ELISA

Antibody responses to *C. perfringens* ε toxin in vaccinated calves are shown in Fig. 2B. Following vaccination, antibody titers increased significantly from baseline, with the highest titers observed on day 42. Antibody titers to *C. perfringens* ε toxin were not significantly different between days 0 and 14 (*P* = 0.3862) or between days 0 and 21 (*P* = 0.1633), indicating that by day 14 and day 21 titers had not significantly increased following the priming dose of vaccine. However, antibody concentrations were significantly different between day 21 and day 42 (*P <* 0.0001), and between day 21 and day 56 (*P* = 0.0228), indicating significant increase following booster. Antibody concentrations were also significantly different between day 0 and day 42 (P *<* 0.0001) and also between day 0 and day 56 (*P* = 0.0006), indicating that priming plus booster dose together induced a significant increase in antibody titer over baseline, measurable both at day 42 and day 56.

### 3.3. Development and optimization of ELISAs for antibodies to clostridial toxin antigens

The ELISA assays for tetanus toxoid (Fig. S1), *C. septicum* α toxin (Fig. S2), *C. novyi* type B toxins (Fig. S3), and *C. sordellii* toxins (Fig. S4) were developed and optimized using microfluidic multiwell microplates. The optimized conditions for each ELISA were as follows: 25 ng/well for coating with tetanus toxoid and 1: 20 dilutions for coating with the other clostridial toxins, 1:50 dilution of serum samples, and 1:80,000 dilution of conjugated secondary antibody for tetanus toxoid and 1:20,000 dilution of the conjugated secondary antibody for the other clostridial antigens. These optimized conditions, summarized in Table 1, were used for all subsequent ELISA assays.

### 3.4. Intra-and inter-assay CV and LOD of optimized microfluidic ELISAs

In order to assess assay repeatability and reproducibility, the CV values were calculated for pooled positive and negative control sera tested in triplicate wells on the same day (intra-assay), as well as for the same assay repeated on different days (inter-assay) and are summarized in Table 2. The assay for antibody to tetanus toxoid showed very low inter-assay and intra-assay CVs (both *<*1 % for intra and ~ 2 % for inter), indicating high repeatability both within and across different assays. The assay for antibody to *C. sordellii* toxins showed the highest intra- and inter-assay CVs for all the assays, with intra-assay CVs of 6.6 % (positive control) and 3.4 % (negative control) and inter-assay CVs of 8.5 % (positive control) and 7.3 % (negative control).

The LOD cut-off for IgG antibody titers against each clostridial toxin antigen was calculated by adding three times the standard deviation (SD) of the pooled negative control serum to the mean of the pooled negative control serum. Both inter- and intra-assay LOD cut-off for IgG antibody titers against each clostridial toxin antigen was calculated and summarized in Table 2. In all cases, the inter-assay LOD values were higher than the intra-assay values, indicating a greater variation across different testing days. Therefore, to determine the corresponding serum dilution, we used the inter-assay LOD values as the cut-off. The results are as follows (Fig. S5): for tetanus toxoid, the inter-assay LOD was 6982.5 fluorescence intensity units, corresponding to a 1:800 serum dilution; for *C. septicum* α toxin, it was 8899.0 fluorescence intensity units, corresponding to a 1:300 dilution; for *C. novyi* type B toxin, it was 6433.5 fluorescence intensity units, corresponding to a 1:800 dilution; and for *C. sordellii* toxins, it was 9445.4 fluorescence intensity units, corresponding to a 1:150 dilution.

### 3.5. Measurement of antibody titers to tetanus toxoid using a microfluidic microplate ELISA

**Fig. 3.**
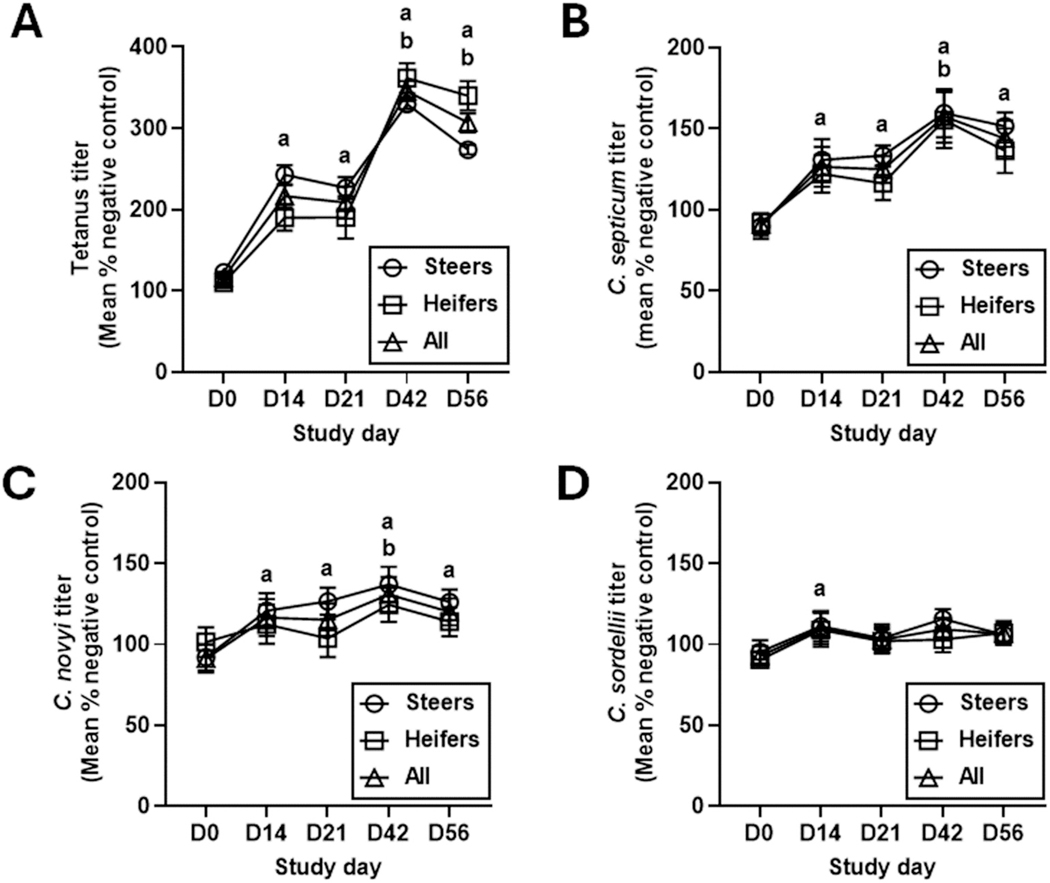
Measurement of IgG titers against (A) tetanus toxoid, (B) *Clostridium septicum* α toxin, (C) *Clostridium novyi* B toxin, and (D) *Clostridium sordellii* toxin in serum from vaccinated calves with a multivalent clostridial vaccine using microfluidic ELISA. Data were expressed as mean percent of the negative control serum when negative control was set as 100 %. The superscript ‘a’ indicates statistically significant differences (*p* < 0.05) on days 14, 21, 42, and 56, compared to day 0, while the superscript ‘b’ denotes significant differences on days 42 and 56, compared to day 21. Statistical significance was evaluated by separate linear mixed models.

The two positive control samples measured 358 % and 444 % relative to the negative control, which was set to 100 %. Antibody responses to tetanus toxoid by vaccinated calves are shown in Fig. 3A. Following vaccination, antibody titers increased significantly from baseline, with the highest titers measured on day 42, similar to the titer of the positive control sample. Unlike *C. perfringens* ε toxin, antibody titers against tetanus toxoid on day 14 were approximately twice as high as day 0 titers. Antibody titers to tetanus toxoid were significantly increased between day 0 and days 14 (*P <* 0.0001) and 21 (*P* = 0.0027), indicating serum antibody concentrations increased significantly after the first dose of vaccine. Antibody titers also showed significant differences between day 0 and day 42 (P *<* 0.0001) and day 0 and day 56 (P *<* 0.0001), as well as between day 21 and day 42 (P *<* 0.0001) and between day 21 and day 56 (*P* = 0.0045), indicating a significant increase in titers following both the priming and booster doses. The booster dose particularly resulted in a substantial increase in antibody titer against tetanus toxoid, with titers more than 3 times higher antibody titers compared before vaccination.

### 3.6. Measurement of antibody titers to C. septicum using a microfluidic microplate ELISA

The two positive control samples measured 256 % and 316 % relative to the negative control, which was set to 100 %. Following the first vaccination, antibody titers to *C. septicum* α toxin significantly increased between day 0 and each of the other days (*P <* 0.0001) with the peak titer observed on day 42, indicating that both the first vaccination dose and booster doses increased the serum antibody titers against *C. septicum* α toxin (Fig. 3B). This trend is very similar to those observed for tetanus toxoid. Although the magnitude of increase is relatively lower, as the highest titers on day 42 did not reach the range of the positive serum control, significant differences were detected between days 21 and 42 (at the peak titer) (*P <* 0.0001) but not between days 21 and 56 (*P* = 0.0642), suggesting a significant transient increase in antibody titers against *C. septicum* α toxin following the booster dose.

### 3.7. Measurement of antibody titers to C. novyi using a microfluidic microplate ELISA

The two positive control samples measured 337 % and 402 % compared to the negative control, which was set to 100 %. Overall, the antibody titers for *C. novyi* type B toxin showed a similar trend to those observed for tetanus toxoid and *C. septicum* α toxin, but the magnitude of increase in antibody titers was much lower than those observed for these two antigens (Fig. 3C). After vaccination, serum antibody titers to *C. novyi* type B toxin in vaccinated calves significantly increased compared to calves before vaccination (day 0), with the highest titer measured on day 42. Antibody titers to *C. novyi* type B toxin were significantly different between day 0 and days 14 (P *<* 0.0001), between day 0 and 21 (*P* = 0.0047), between day 0 and day 42 (P *<* 0.0001), and between day 0 and day 56 (*P* = 0.0033), indicating a significant increase after the first vaccination. However, there was also a significant increase in titer from day 21 to day 42 (*P* = 0.0303), but no significant difference was detected in antibody titers against *C. novyi* type B toxin between day 21 and day 56 (*P* = 0.9170). This suggests that although the first vaccination induced a significant increase in antibody titer over the baseline titer, there was only a transitory booster effect on antibody titers.

### 3.8. Measurement of antibody titer to C. sordellii using a microfluidic microplate ELISA

The two positive control samples measured 154 % and 171 % of the negative control, which was set to 100 %. Therefore, compared to antibody titers against the other clostridial antigens in the positive control samples, the *C. sordellii* toxins titers were notably lower, only 1.5–1.7 times higher than the negative control (Fig. 3D). Similarly, in vaccinated calves, antibody responses to *C. sordellii* toxins appeared to be weaker than those observed for other clostridial antigens. A significant difference was observed only between days 0 and 14 (*p* = 0.0028), indicating that antibody titers were significantly increased after the first vaccination but were not significantly elevated past day 14. Moreover, no significant increase was detected after the booster doses.

## 4. Discussion

Despite the availability of multivalent clostridial vaccines for cattle, assays for measuring IgG titers to these vaccines remain limited. At the time this study began, available commercial ELISA kits only targeted *Clostridium perfringens* toxins, and these required high sample and reagent volumes (50–100 μL per reaction), prolonged incubation times (4 h to overnight), and high costs, limiting their feasibility for large-scale studies of the response of cattle to vaccination.

This study has resulted in the development and optimization of a microfluidic ELISA for measuring IgG antibody titers against clostridial toxins in cattle vaccinated with a multivalent clostridial vaccine. The optimized microfluidic ELISAs demonstrated high repeatability and reproducibility, with intra- and inter-assay coefficient of variation (CV) values below 10 % for all tested clostridial antigens in control sera. This assay offers significant improvements over traditional ELISA methods, including reduced sample volume (5 μL per sample), fast processing time (*<* 1 h), and lower reagent consumption.

The microfluidic ELISA plates utilize a spiral microfluidic channel, providing a 1.5-fold increase in surface area and a 50-fold higher surface-area-to-volume ratio compared to conventional ELISA plates (Kai et al., 2012). This innovative design enhances antigen-antibody interactions by maximizing the reaction surface, leading to accurate and rapid results (Kai et al., 2012; Lee et al., 2020). Due to these advantages, microfluidic technology is continuously being developed for application in various immunoassay platforms beyond ELISA (Berlanda et al., 2021; Shi et al., 2021; Wu et al., 2022). Additionally, the absorbent pad beneath the plates enables a flow-through mechanism, allowing reagents to be added without manual well-emptying (Fig. 1A). This streamlines the assay workflow and significantly reduces processing time, particularly during the washing steps, making the assay more efficient than conventional ELISA plates. In this study, the microfluidic platform successfully detected IgG titers in vaccinated cattle using only 5 μL of diluted serum (1:50) and clostridial toxin antigens. The optimized incubation times for most steps were 5–10 min, with the final substrate development taking 15 min, resulting in a total assay time of *<*1 h. The ability to achieve reliable assay performance in such a short time frame suggests that this assay allows for rapid and sensitive antigen-antibody detection. In our study, to optimize assay conditions, various serum and antigen dilution factors were tested. The limit of detection (LOD) of IgG titers was determined using pooled negative control sera (1:50 diluted) under optimized ELISA conditions. The LOD for IgG titers against each antigen was found to be as follows (Fig. S5): tetanus toxoid at 1:800, *C. septicum* α toxin at 1:300, *C. novyi* at 1:800, *C. sordellii* and at 1:150. These dilution factors represent the lowest concentration at which antibody titers could be reliably detected. For comparison, the commercial ELISA for *C. perfringens* ε toxin require we used 1:1 diluted serum sample to measure the antibody titers, suggesting that our microfluidic ELISA is highly sensitive for detecting IgG titers from serum samples. Our results align with previous research on the use of microfluidic microplates with spiral channels for biomarker detection, including a study by Lee et al. (2020) that demonstrated the high sensitivity and specificity of microfluidic microplates for detecting *Plasmodium falciparum* biomarkers in malaria-infected patient serum. Overall, the microfluidic ELISA is a cost-effective, sensitive, reproducible, and practical tool for antibody detection and herd-level analyses in cattle.

Our findings confirmed that the multivalent clostridial vaccine effectively activated humoral responses in cattle, as IgG titers significantly increased after vaccination for most clostridial antigens, as measured using our optimized microfluidic ELISA. Specifically, IgG antibody titers against all tested clostridial antigens showed either substantial (*C. perfringens* ε toxin) or significant increases by day 14 following the initial vaccination (Figs. 2B and 3). Furthermore, the booster dose on day 21 significantly enhanced IgG titers for all clostridial toxin antigens, except *C. sordellii* toxins, by day 42. These results are consistent with previous studies (Woolums et al., 2011), which also demonstrated robust humoral responses to *C. perfringens* β toxin, *C. novyi* α toxin, and *C. septicum* α toxin in cattle vaccinated with multivalent clostridial vaccines, as measured using conventional in-house ELISA. Interestingly, a notable discrepancy was observed in responses to *C. sordellii*, with previous work identifying significantly higher toxin-neutralizing titers for *C. sordellii* after both primary and booster vaccinations (Woolums et al., 2011), whereas in our study, titers for this antigen remained low, similar to levels observed in the negative control serum (Fig. 3D). This discrepancy may be due to methodological differences, as the previous study used *Clostridium difficile* toxin B as a surrogate in a cytotoxicity assay to measure neutralizing antibodies against *C. sordellii*, while we directly measured antigen-specific IgG titers using ELISA. These differences underscore the impact of assay methodology and antigen specificity on the evaluation of humoral responses to multivalent vaccines.

Despite the promising performance of microfluidic ELISA in this study, a few aspects could be improved. First, we used culture filtrates of *C. novyi, C. septicum*, and *C. sordellii* obtained from USDA-APHIS as antigens, which likely contributed to the relatively lower IgG titers compared to commercially purified tetanus toxoid. The use of recombinant antigens might significantly enhance the sensitivity of the microfluidic ELISA to detect IgG titers for specific clostridial species, as they have for traditional ELISAs (Woolums et al., 2011). Second, while the multivalent clostridial vaccine contained the toxin of *Clostridium chauvoei,* which causes necrotizing myositis and fasciitis (“blackleg”) in cattle, we were unable to evaluate IgG responses to this agent due to the unavailability of its toxin antigens. Thus, there may be a need to optimize and test microfluidic ELISA for *C. chauvoei* antigens, as well as other untested clostridial components, in future studies. Last, it will be necessary to test this microfluidic ELISA on a larger number of samples from cattle vaccinated with multivalent clostridial vaccines to confirm the value of this microfluidic ELISA method for use in field trials.

While we cannot definitively say that the serum antibody titers measured following vaccination of the cattle in this study would have protected them against disease, the assays described here could be used in future field trials to evaluate the relationship between humoral immunity and disease incidence. To determine the functional significance of these antibodies, comparison with *in vitro* and *in vivo* toxin neutralization assays will be necessary. However, previous studies have demonstrated a strong correlation between antibody titers measured by ELISA and results of toxin neutralization assays. A significant correlation has been reported between ELISA and *in vitro* toxin neutralization assays for *C. perfringens* epsilon, *C. tetani*, *C. septicum*, and *C. novyi* (*r* = 0.84–0.94, *p <*0.001) (Wood, 1991). Similarly, a strong correlation with *in vivo* toxin neutralization assays in mice for antibodies to tetanus toxoid (*r* = 0.94, p *<* 0.001) was found (Manghi et al., 1994), highlighting the potential of ELISA as an alternative measure of immunity. While these studies support the utility of ELISA-based approaches, further validation is required to establish a similar correlation for the microfluidic ELISA developed in this study. Nonetheless, this assay offers a rapid, cost-effective alternative to conventional methods. This microfluidic ELISA could prove to be a useful tool for evaluating humoral responses to multivalent clostridial vaccines in cattle, particularly for herd-level monitoring. With further refinement and validation, this assay could support improved vaccine evaluation and development of better disease prevention strategies.

## Supplementary Material

1

## Figures and Tables

**Table 1:** Optimized titration of antigens, serum, and secondary antibody.

Clostridial antigen	Antigen coating concentration or dilution	Serum dilution	Secondary antibody dilution
Tetanus toxoid	5 μg/mL	1:50	1:80,000
*C. septicum α* toxins	1:20	1:50	1:20,000
*C. novyi* type B toxins	1:20	1:50	1:20,000
*C. sordellii* toxins	1:20	1:50	1:20,000

**Table 2:** Intra- and inter-assay variation, and limit of detection (LOD) in the optimized microfluidic ELISA.

Antigens	Assay	Pooled positive control serum			Pooled negative control serum			
		Mean	SD	CV (%)	Mean	SD	CV (%)	LOD cut-off
Tetanus toxoid	Intra-	32,232.0	40.1	0.1	6859.3	32.0	0.5	6955.3
	Inter-	31,070.3	595.3	1.9	6878.9	34.5	0.5	6982.5
*C. septicum* α toxins	Intra-	31,643.0	131.7	0.4	7436.1	247.6	3.3	8178.8
	Inter-	30,054.0	699.3	2.3	7841.8	352.7	4.5	8899.0
*C. novyi* type B toxins	Intra-	25,802.7	990.3	3.8	6002.0	120.2	2.0	6362.7
	Inter-	25,274.0	1480.2	5.9	5773.4	220.0	3.8	6433.5
*C. sordellii* toxins	Intra-	16,439.0	1090.4	6.6	7395.7	253.3	3.4	8155.7
	Inter-	16,517.7	1405.7	8.5	7751.4	564.7	7.3	9445.4

The repeatability and reproducibility of each ELISA were evaluated using pooled positive and negative control sera under the optimized conditions. Sera were tested in triplicate on the same day (intra-assay) and over three different days (inter-assay) using the optimized microfluidic ELISA. The mean represents the average fluorescence intensity, SD refers to the standard deviation, and CV denotes the coefficient of variation (%).

## Data Availability

Data will be made available on request.

## Appendix A. Supplementary data

Supplementary data to this article can be found online at https://doi.org/10.1016/j.jim.2025.113900.
