# Supplementary material for: Development of microfluidic ELISA for measuring humoral responses to clostridial antigens in vaccinated cattle
Source: J Immunol Methods. Author manuscript; Available in PMC 2026 Jun 10. (PMC13252714; doi:10.1016/j.jim.2025.113900)
Supplement: 1 [file NIHMS2168621-supplement-1.docx]

**
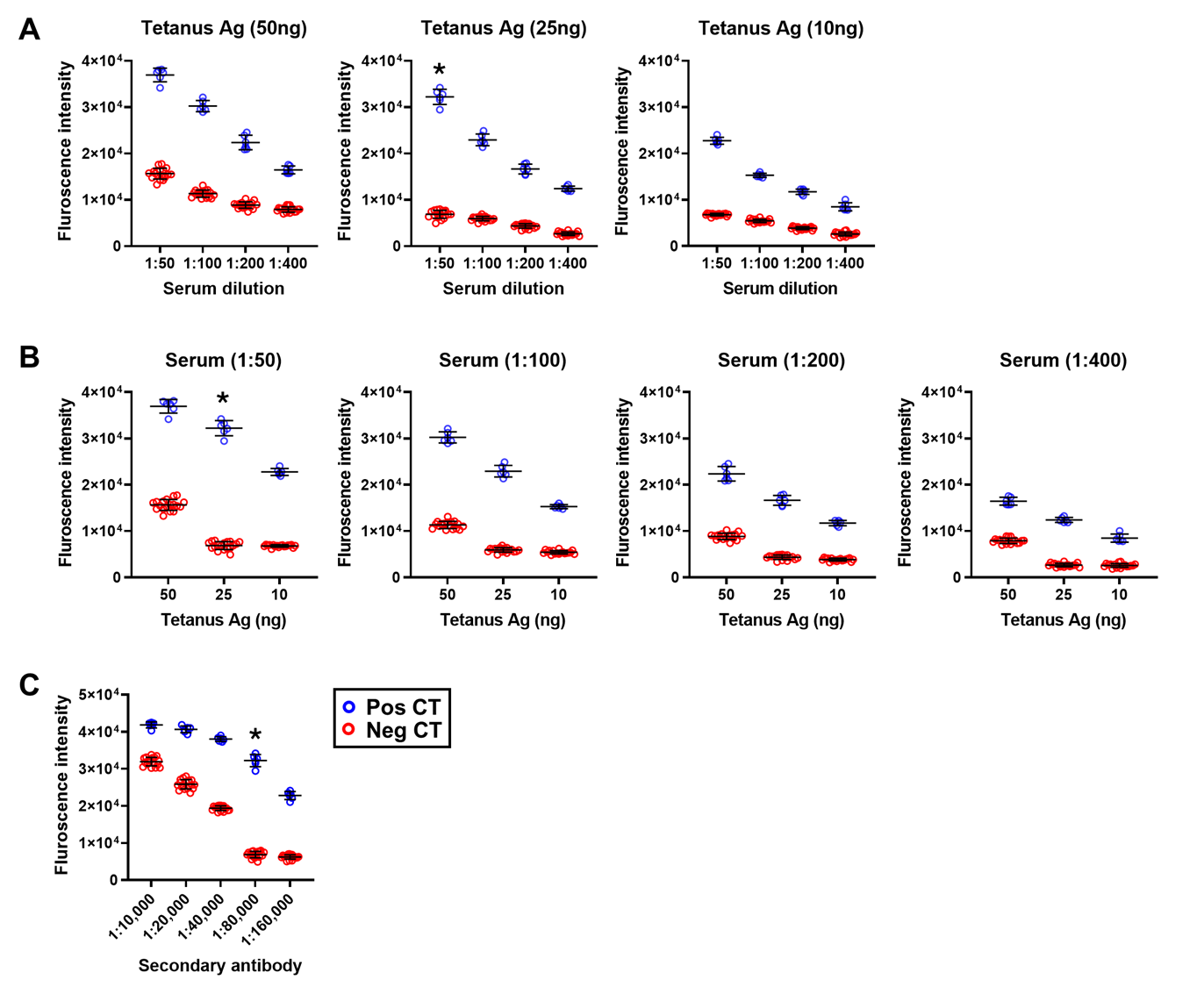
Supplementary figures.**

**Figure S1. Optimization of ELISA for tetanus toxoid using microfluidic multiwell microplate.**

(A) and (B) plates were coated with different amounts of tetanus toxoid. After blocking and PBS wash, positive and negative control serum samples were serially diluted (as indicated) and added to the plate. After a 5-min incubation at room temperature (RT), horseradish peroxidase (HRP) conjugated sheep anti-bovine IgG1 was added to the plate and incubated for 5 min at RT. After PBS wash, HRP enhancer was added and incubated for 15 min at RT. The florescence readings were measured at 530 nm for excitation and 590 nm for emission. (C) Plates were coated with 25 ng/well of tetanus toxoid, and serum samples were used at 1:50 dilution, and conjugated secondary antibody dilutions ranging from 1:10,000 to 1:160,000 (2-fold serial dilution). Each sample was tested in triplicate. Results were expressed as mean ± SD of the fluorescence readings. Asterisks (*) indicate the highest ratio of fluorescence intensity from positive to negative serum samples.

**
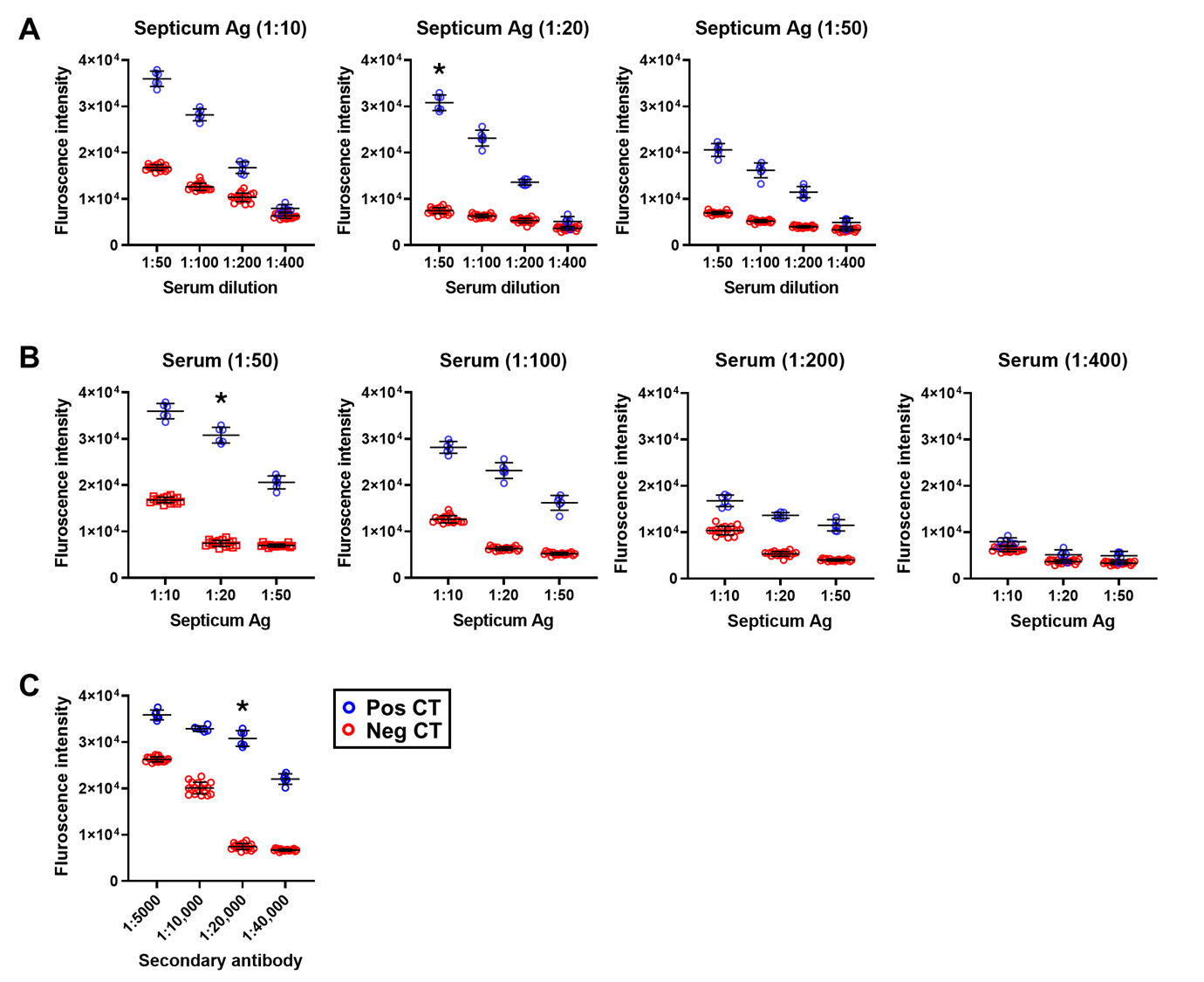
Figure S2. Optimization of ELISA for *Clostridium septicum* α toxin using microfluidic multiwell microplate.**

(A) and (B) plates were coated with different amounts of *C. septicum* α toxin. After blocking and PBS wash, positive and negative control serum samples were serially diluted (as indicated) and added to the plate. After a 5-min incubation at room temperature (RT), horseradish peroxidase (HRP) conjugated sheep anti-bovine IgG1 was added to the plate and incubated for 5 min at RT. After PBS wash, HRP enhancer was added and incubated for 15 min at RT. The florescence readings were measured at 530 nm for excitation and 590 nm for emission using Cytation5 microplate reader. (C) Plates were coated with 1:20 dilution of *C. septicum* α toxins, and serum samples were used at 1:50 dilution, and conjugated secondary antibody dilutions ranging from 1:5,000 to 1:40,000 (2-fold serial dilution). Each sample was tested in triplicate. Results were expressed as mean ± SD of the fluorescence readings. Asterisks (*) indicate the highest ratio of fluorescence intensity from positive to negative serum samples.

**
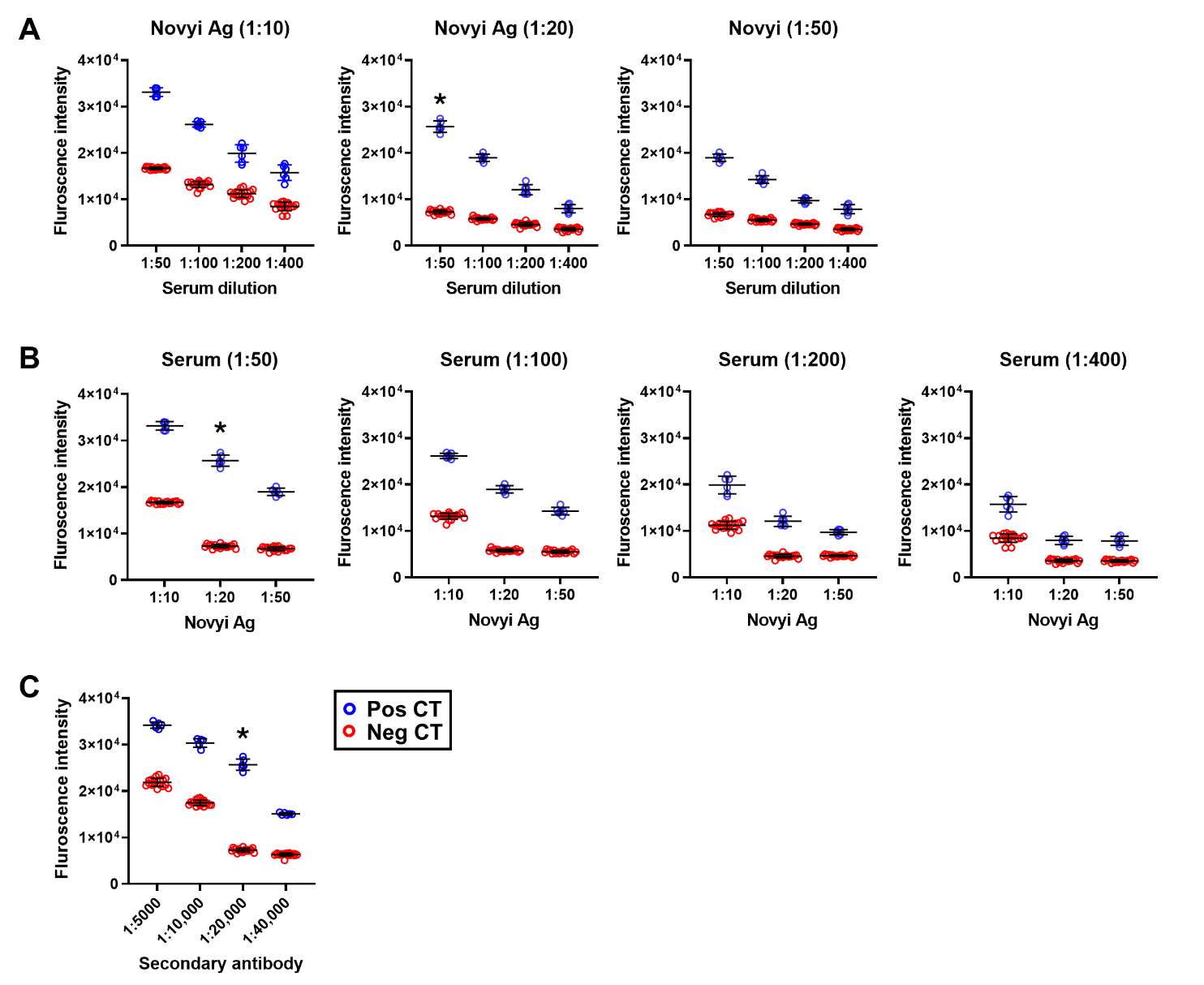
Figure S3. Optimization of ELISA for *Clostridium novyi* B toxin using microfluidic multiwell microplate.**

(A) and (B) plates were coated with different dilutions of *C. novyi* B toxin. After blocking and PBS wash, positive and negative control serum samples were serially diluted (as indicated) and added to the plate. After a 5-min incubation at room temperature (RT), horseradish peroxidase (HRP) conjugated sheep anti-bovine IgG1 was added to the plate and incubated for 5 min at RT. After PBS wash, HRP enhancer was added and incubated for 15 min at RT. The florescence readings were measured at 530 nm for excitation and 590 nm for emission using Cytation5 microplate reader. (C) Plates were coated with 1:20 dilution of *C. novyi* B toxins, and serum samples were used at 1:50 dilution, and conjugated secondary antibody dilutions ranging from 1:5,000 to 1:40,000 (2-fold serial dilution). Each sample was tested in triplicate. Results were expressed as mean ± SD of the fluorescence readings. Asterisks (*) indicate the highest ratio of fluorescence intensity from positive to negative serum samples.

**
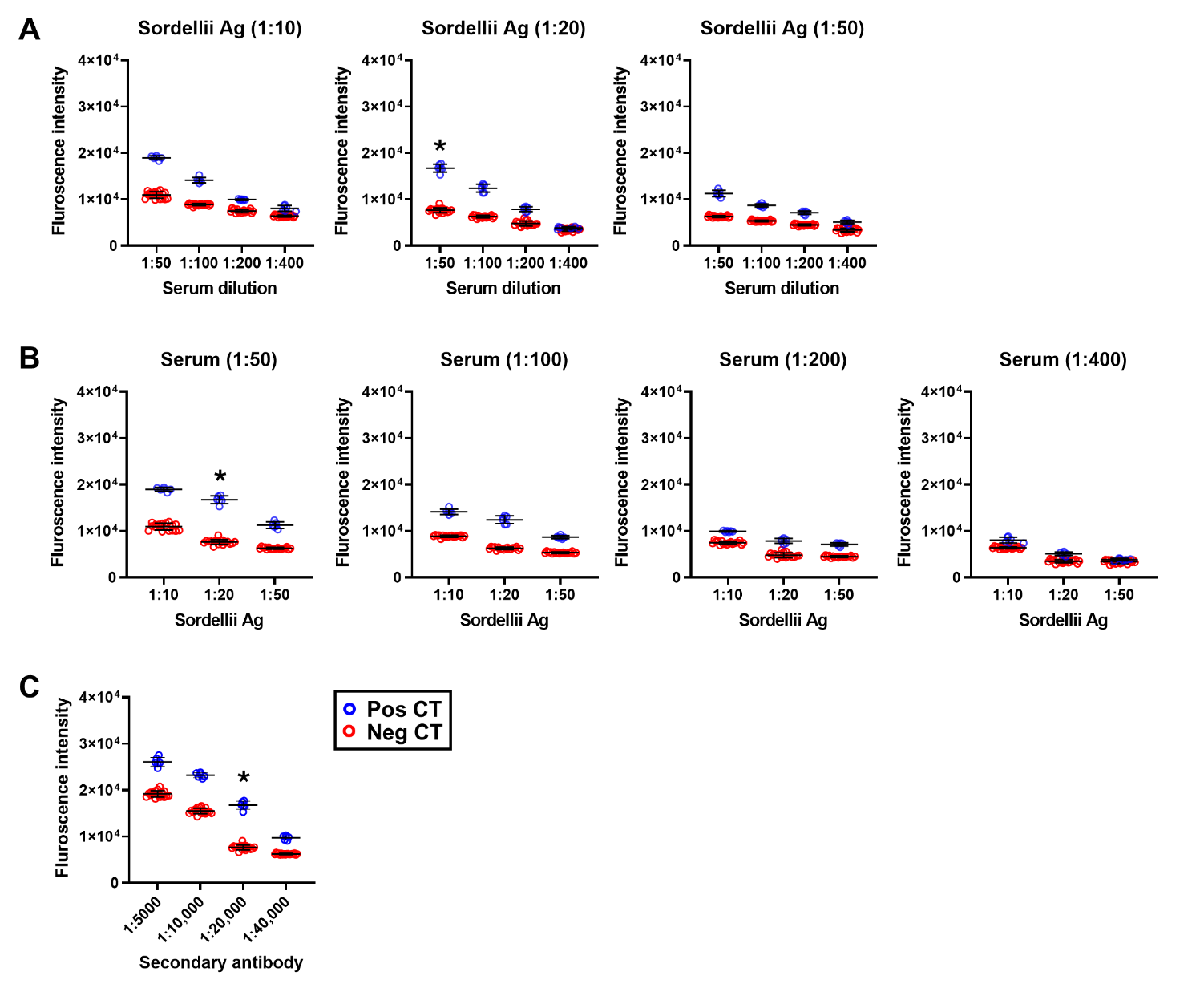
Figure S4. Optimization of ELISA for *Clostridium sordellii* toxin using microfluidic multiwell microplate.**

(A) and (B) plates were coated with different dilutions of *C. sordellii* toxin. After blocking and PBS wash, positive and negative control serum samples were serially diluted (as indicated) and added to the plate. After a 5-min incubation at room temperature (RT), horseradish peroxidase (HRP) conjugated sheep anti-bovine IgG1 was added to the plate and incubated for 5 min at RT. After PBS wash, HRP enhancer was added and incubated for 15 min at RT. The florescence readings were measured at 530 nm for excitation and 590 nm for emission using Cytation5 microplate reader. (C) Plates were coated with 1:20 dilution of *C. sordellii* toxins, and serum samples were used at 1:50 dilution, and conjugated secondary antibody dilutions ranging from 1:5,000 to 1:40,000 (2-fold serial dilution). Each sample was tested in triplicate. Results were expressed as mean ± SD of the fluorescence readings. Asterisks (*) indicate the highest ratio of fluorescence intensity from positive to negative serum samples.

**
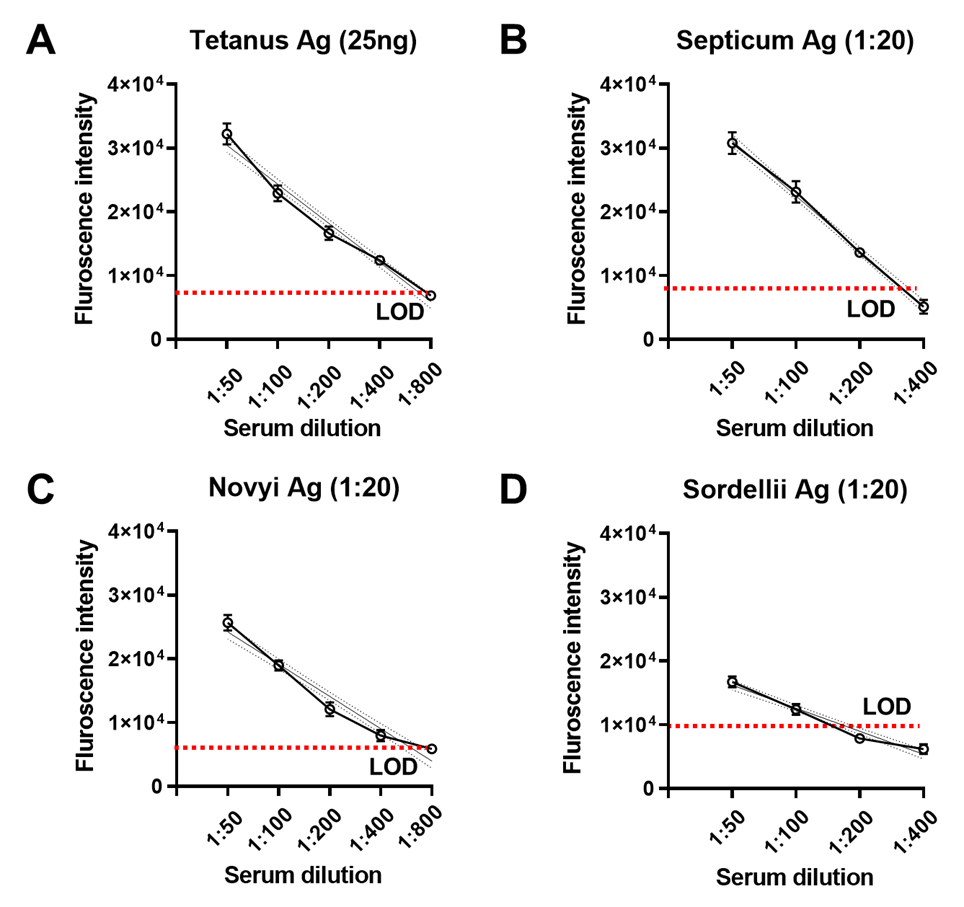
**

**Figure S5. The limit of detection (LOD) determination for IgG titers against each clostridial antigens.**

The LOD for IgG titers against each clostridial antigen was calculated to assess the analytical sensitivity of each assay. Pooled positive control serum was subjected to 2-fold serial dilutions within the indicated range on the graph. The LOD cut-off value for IgG titers against each clostridial antigen for IgG titers against each clostridial antigen was determined based on the mean fluorescence intensity and standard deviation (SD) of the pooled negative control sera (1:50 diluted). Specifically, the LOD was defined as the lowest antibody dilution at which the fluorescence intensity of the positive control serum exceeded the mean fluorescence intensity of the negative control by at least 3×SD. The LOD cut-off values for IgG titers against (A) tetanus toxoid, (B) *C. septicum* α toxins (C) *C. novyi* type B toxins, and (D) *C. sordellii* toxins are indicated by the red dotted line. For tetanus toxoid, the LOD for fluorescence intensity was 6982.5, corresponding to a 1:800 serum dilution; for *C. septicum* α toxin, it was 8899.0 fluorescence intensity, corresponding to a 1:300 dilution; for *C. novyi* type B toxin, it was 6433.5 fluorescence intensity, corresponding to a 1:800 dilution; and for *C. sordellii* toxins, it was 9445.4 fluorescence intensity, corresponding to a 1:150 dilution.
